# Evaluation of a CD13 and Integrin α_v_β_3_ Dual-Receptor Targeted Tracer ^68^Ga-NGR-RGD for Ovarian Tumor Imaging: Comparison With ^18^F-FDG

**DOI:** 10.3389/fonc.2022.884554

**Published:** 2022-05-18

**Authors:** Yu Long, Fuqiang Shao, Hao Ji, Xiangming Song, Xiaoying Lv, Xiaotian Xia, Qingyao Liu, Yongxue Zhang, Dexing Zeng, Xiaoli Lan, Yongkang Gai

**Affiliations:** ^1^ Department of Nuclear Medicine, Union Hospital, Tongji Medical College, Huazhong University of Science and Technology, Wuhan, China; ^2^ Hubei Province Key Laboratory of Molecular Imaging, Wuhan, China; ^3^ Department of Radiology, University of Pittsburgh, Pittsburgh, PA, United States

**Keywords:** positron emission tomography (PET), ovarian cancer, CD13, integrin α_v_β_3_, dual-receptor targeted

## Abstract

Ovarian cancer has the highest mortality rate of gynecologic malignancy. ^18^F-FDG positron emission tomography (PET) adds an important superiority over traditional anatomic imaging modalities in oncological imaging but has drawbacks including false negative results at the early stage of ovarian cancer, and false positives when inflammatory comorbidities are present. Aminopeptidase N (APN, also known as CD13) and integrin α_v_β_3_ are two important targets overexpressed on tumor neo-vessels and frequently on ovarian cancerous cells. In this study, we used subcutaneous and metastatic models of ovarian cancer and muscular inflammation models to identify ^68^Ga-NGR-RGD, a heterodimeric tracer consisting of NGR and RGD peptides targeting CD13 and integrin α_v_β_3_, respectively, and compared it with ^18^F-FDG. We found that ^68^Ga-NGR-RGD showed greater contrast in SKOV3 and ES-2 tumors than ^18^F-FDG. Low accumulation of ^68^Ga-NGR-RGD but avid uptake of ^18^F-FDG were observed in inflammatory muscle. In abdominal metastasis models, PET imaging with ^68^Ga-NGR-RGD allowed for rapid and clear delineation of both peritoneal and liver metastases (3-6 mm), whereas, ^18^F-FDG could not distinguish the metastasis lesions due to the relatively low metabolic activity in tumors and the interference of intestinal physiological ^18^F-FDG uptake. Due to the high tumor-targeting efficacy, low inflammatory uptake, and higher tumor-to-background ratios compared to that of ^18^F-FDG, ^68^Ga-NGR-RGD presents a promising imaging agent for diagnosis, staging, and follow-up of ovarian tumors.

## Introduction

Ovarian cancer has the highest mortality rate of all gynecologic malignant cancers, with more than 80% of patients presenting with advanced disease (1). Due to their silent nature of the disease, patients often present with advanced stages at first diagnosis, which will result in 29-75% of patients succumbing to ovarian cancer within 5 years. However, if diagnosed at stage I (ovary defined), the 5-year survival rate exceeds 90% (1, 2). Therefore, it’s a research priority to improve early detection and prevention, as a better prognosis correlated with early stage at diagnosis.

Functional imaging plays an essential role in the management of ovarian cancers. In particular, with the development and promotion of PET/MRI (3, 4) with excellent soft tissue contrast and digital PET scanner (5, 6) with higher sensitivity and diagnostic performance than analog PET, functional imaging will reduce radiation dose, enhance the diagnostic confidence and ensure the better strategies for patient management and personalized treatment, showing a wider clinical application prospect. ^18^F-FDG positron emission tomography/computed tomography (PET/CT) imaging, as the most frequently used functional imaging method in oncological imaging, adds an important superiority over traditional anatomic imaging modalities by providing functional information about cellular glucose metabolism. However, ^18^F-FDG PET is not recommended for the primary detection of ovarian cancers with a relatively low level of sensitivity (52-58%) and specificity (76-78%) (1, 7, 8), which might be due to tumor size and cystic or mucinous histological features with no/low metabolic activity in tumors (9, 10). Besides, it is limited by several pitfalls, such as higher ovarian glucose metabolism during menstruation and midcycle, physiologic accumulation in several benign diseases, as well as its imprecise distinction between cancerous growths and acute inflammation lesions (11–14). Novel PET agents targeting biological tumor features, including cell proliferation, angiogenesis, hypoxia, metabolism, and receptor overexpression, are pursued in preclinical researches to better detect early malignant lesions, evaluate the heterogeneity of biological features, and monitoring treatment response more accurately (10, 15–19).

Angiogenesis plays a prominent role in tumor growth, invasion and metastasis by providing abundant oxygen, nutrients, and metastatic conduits (20). aminopeptidase N (APN, also known as CD13) and integrin α_v_β_3_ are two key regulators involved in tumor angiogenesis and tumor progression. They are overexpressed on the tumoral neo-endothelial cells during angiogenesis as well as cancerous cells, regarded as two important hallmarks of tumor angiogenesis (21–24). There are several studies have focused on the imaging and/or treatment of ovarian tumors by targeting CD13 (25, 26) or integrin α_v_β_3_ (27, 28). However, these angiogenesis-related factors, including CD13 and integrin α_v_β_3_, are usually differentially expressed in ovarian tumor tissues and cell lines due to the heterogeneity and genetically instability of the disease (29, 30), making it a very challenging approach to find “the optimal target”, and may also be one of the reasons for drug resistance of cancer to monotherapy. Therefore, an alternative approach is to develop a complementary receptor-targeting agent for the detection and treatment of tumors.

Previously, we developed a CD13 and integrin α_v_β_3_ dual-receptor targeted radiotracer, ^68^Ga-NGR-RGD, which demonstrated promising results in PET imaging of breast cancers with superior imaging efficacy than monomeric ^68^Ga-NGR and ^68^Ga-RGD (31). Furthermore, the physiological uptake of ^68^Ga-NGR-RGD is low in most normal organs, except kidneys, which may make this dual-receptor targeted tracer supplement or even be superior to ^18^F-FDG PET/CT in the early diagnosis and staging of ovarian tumors. In this study, we aim to evaluate the value of ^68^Ga-NGR-RGD in PET/CT imaging of ovarian tumors. In addition, we also investigated its potential application in distinguishing tumors and inflammation. Routine ^18^F-FDG imaging was also conducted as a control group in all prepared mice models.

## Materials and Methods

### Synthesis of NGR-RGD and Radiolabeling

NGR-RGD was synthesized and radiolabeled using our previously developed method (31). Briefly, 150 µL sodium acetate buffer (0.25 M, pH 6.8) and 2 µL NGR-RGD (2 mM) were added to the tube containing 500 µL ^68^GaCl_3_ in 0.05 M HCl (150-200 MBq) and mixed. The final pH of the radiolabeling solution was approximately 4.0. Then, the mixture was heated at 95°C for 5 min. The radiolabeling field of the product ^68^Ga-NGR-RGD was determined by radio-HPLC. ^68^Ga was produced with a ^68^Ge/^68^Ga generator (Isotope Technologies Garching GmbH, Garching, Germany). Peptides were obtained commercially from Chinapeptide (Shanghai, China) or Gl Biochem (Shanghai, China).

### Cell Culture

Human ovarian cancer cells, SKOV3, ES-2, and OVCAR4 were derived from our own laboratory preservation and cultured in GibcoDulbecco’s Modified Eagle Medium/Nutrient mixture F-12 (DMEM/F12; Gibco, Carlsbad CA, USA), supplemented with 10% fetal bovine serum (FBS; Sciencell, Carlsbad CA, USA), 100 mg/mL streptomycin and 100 mg/mL penicillin (Solarbio, Shanghai, China) at 37°C in a humidified incubator with 5% CO_2_.

### Western Blot Analysis

Cancer cells were harvested, and total protein concentration was measured with the BCA protein assay kit (Aidlab, Beijing, China). After denaturation and separation by SDS-PAGE, proteins were transferred to a polyvinylidenefluoride (PVDF) membrane. Next, the blots were incubated with primary antibodies (1:500 anti-CD13, 1:1000 anti-Integrin alpha V, and 1:1000 anti-Integrin beta 3; Abcam, Cambridge MA, USA), and Glyceraldehyde-3-phosphate dehydrogenase (1:10000 GAPDH; Sungene, Tianjin, China). Next, the membrane was incubated with goat anti-rabbit IgG/HRP (diluted 1:20000; Sungene, Tianjin, China). The membrane was scanned by enhanced chemiluminescence (ECL kit, Beyotime) and analyzed using Quantity One software (Bio-Rad, Hercules CA, USA).

### *In Vitro* Cell Uptake and Blocking Studies

Cells in logarithmic phase were harvested and counted using a cytometer (Cellmeter Mini, Nexcelom Bioscience LLC, Lawrence MA, USA). Cells were seeded in a 24-well plate at 2×10^5^ cells per well 24 h in advance. 74 kBq ^68^Ga-NGR-RGD in 50 µL PBS were added to each well and incubated at 37°C for 30 min, 1 h and 2 h. For blocking study, cells were pretreated with one hundred times excess of non-radioactive NGR-RGD or NGR + RGD 15 min in advance. At the end of each time point, supernatant was collected, and cells were washed twice with pre-cooled PBS (wash 1) before lysed with 1 N sodium hydroxide; then each well was washed twice with pre-cooled PBS (wash 2). Cells, supernatant and wash solutions were subjected to radioactivity analysis using an automatic gamma counter (2470 WIZARD; PerkinElmer, Waltham MA, USA). The percentage of radioactivity taken up by the cells was calculated according to Equation 1, wherein Cpm represents decay-corrected radioactivity counts per minute.


(Equation 1) % radioactivity uptake=[Cpm(Cells)+Cpm(Wash 2)]/[Cpm(Cells)+Cpm(Supernatant)+Cpm(Wash 1)+Cpm(Wash 2)]×100

### Animal Models

All animal studies were carried out according to the regulations and standards of the Institutional Animal Care and Use Committee of Tongji Medical College of Huazhong University of Science and Technology. Subcutaneous SKOV3 or ES-2 tumors were engrafted into 4-6 weeks-old female BALB/C nude mice obtained from Beijing HFK Bioscience Co. Ltd (Beijing, China). For implantation, 5×10^6^ cancer cells in 100 µL PBS were subcutaneously injected into the right shoulder of each mouse. The mice were subjected to the following experiments when tumor size reached 8-10 mm. For mouse muscular inflammation models, 20 μL turpentine oil (Aladdin, China) was injected into the right thigh muscle of each mouse using a 29-gauge hypodermic needle. Turpentine oil caused visible redness and swelling within 3 h after injection and the inflammation mice were subjected to PET/CT scans at 24 h after injection of turpentine oil.

For abdominal metastasis models, SKOV3 or ES-2 cells were harvested and resuspended in a mixed solution (50% Matrigel, Corning and 50% PBS). Next, 5×10^6^ tumor cells in 200 µL were injected into intra-peritoneal cavity (1, 32, 33). About 20 days later, the mice underwent PET/CT imaging.

### Animal PET/CT Imaging and Biodistribution

PET/CT imaging was performed on the lnliView-3000B small animal PET/SPECT/CT (Novel Medical, Beijing, China). Overnight fasted tumor-bearing and inflammation mice received intravenous (i.v.) injection of 2.4-3 MBq ^18^F-FDG. Animals were then returned to anesthesia induction box and subsequently anesthetized with 2.0% isoflurane delivered in 100% air. PET/CT scans were performed at 1 h after injection. The day after ^18^F-FDG imaging, the PET/CT-based protocol for ^68^Ga-NGR-RGD imaging were conducted, including intravenous injection of the ^68^Ga-NGR-RGD solution (4-5.5 MBq) and identical procedures. For abdominal metastasis groups, PET/CT were performed at 1 h p.i. of ^18^F-FDG or ^68^Ga-NGR-RGD. Images were quantified *via* region-of-interest (ROI) analysis.

Following the terminal PET/CT scan, mice were sacrificed, and organs of interest were harvested, weighed, and γ-counted (2470 WIZARD; PerkinElmer, Waltham MA, USA) to validate the imaging data. The tracer accumulation of tissues and organs were noted by the percentage of injected dose per gram of tissue and corrected for radioactive decay (%ID/g).

### Immunohistochemistry Analysis

Tumors were extracted, fixed in 4% paraformaldehyde, and then dehydrated and embedded in paraffin. Fixed tumor tissue sections (5 μm) were deparaffinized, rehydrated and permeabilized in EDTA buffer (pH 9.0). The sections were blocked for nonspecific binding by adding 3% hydrogen peroxide and 10% normal goat serum. Sections were incubated with primary antibodies at 4°C overnight (anti-α_v_β_3_, 1:100; anti-CD13, 1:100; anti-CD31, 1:2000, Abcam, Cambridge MA, USA). Then sections were further stained with secondary antibody (HRP-labeled goat anti-rabbit IgG, diluted 1:50) at room temperature for 25 min, and then incubated with 3,3’-diaminobenzidine (DAB, Beyotime, Hangzhou, China) for 5 min. Last, slides were counterstained with hematoxylin (Beyotime), dehydrated, covered, and observed under light microscopy.

### Statistical Analysis

Quantitative data were described as the mean ± standard deviation (SD). Statistical analysis was performed using student *t*-test and p-values < 0.05 were considered statistically significant.

## Results

### CD13 and Integrin α_v_β_3_ Expression in Ovarian Tumor Cell Lines

**Figure 1:**
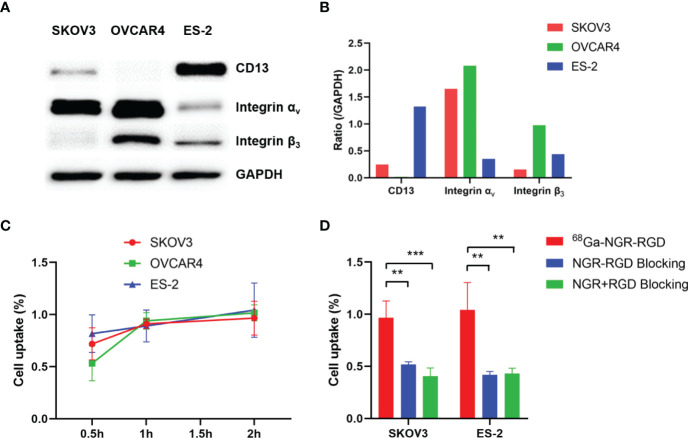
Evaluation of binding affinity of ^68^Ga-NGR-RGD to ovarian tumor cells. **(A)** Western blot analysis of expression of CD13, integrin α_v_ and integrin β_3_ in three ovarian tumor cell lines, with GAPDH used as internal control. **(B)** The semi-quantitative analysis was conducted through the integrated optical density ratio of CD13, integrin α_v_ and integrin β_3_ to GAPDH. **(C)** Uptake of ^68^Ga-NGR-RGD in SKOV3, OVCAR4 and ES-2 ovarian tumor cell lines at 0.5 h, 1 h, 2 h. **(D)** Uptake of ^68^Ga-NGR-RGD in SKOV3 and ES-2 cells with or without blocking dose of NGR-RGD or NRG + RGD at 2 h. Cell uptake and blocking assays showed the ^68^Ga-NGR-RGD displayed specific binding to ovarian tumor cell lines. **p < 0.01, ***p < 0.001. Data are expressed as mean ± SD (n = 4).

Expression levels of CD13 and integrin α_v_β_3_ in three ovarian tumor cell lines were determined *via* Western blot, with GAPDH used as an internal control (**Figures 1A, B**). Strong integrin α_v_β_3_ band intensity was observed in SKOV3 and OVCAR4 cell lines and strong CD13 staining was found in ES-2 cell line, indicating the high expression of CD13 and/or integrin α_v_β_3_ in ovarian tumor cell lines.

### Uptake Profile of ^68^Ga-NGR-RGD in Ovarian Tumor Cells

To demonstrate the specificity of NGR-RGD for ovarian tumor cells, we conducted the cell uptake and blocking studies of ^68^Ga-NGR-RGD in SKOV3, OVCAR4, and ES-2 ovarian tumor cells. High uptake of ^68^Ga-NGR-RGD was observed in these three ovarian tumor cells with a gradually increasing trend over time (**Figure 1C**). On the contrary, minimal uptake of ^68^Ga-NGR-RGD by SKOV3 and ES-2 cells was detected when pretreated with excess amounts of non-radiolabeled NGR-RGD or NGR+RGD (**Figure 1D**).

### PET/CT Imaging and Biodistribution of ^68^Ga-NGR-RGD in Subcutaneous Tumors

**Figure 2:**
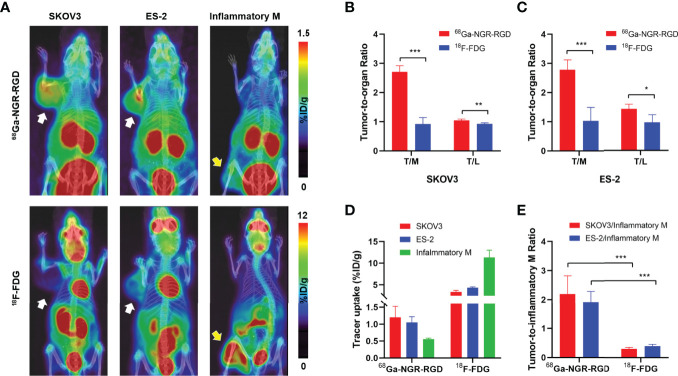
PET/CT imaging and quantitative analysis of ^68^Ga-NGR-RGD and ^18^F-FDG in subcutaneous ovarian cancer models and inflammation models. **(A)** Representative static small PET/CT images of ^68^Ga-NGR-RGD and ^18^F-FDG in SKOV3 and ES-2 xenograft mice and turpentine oil-induced muscular inflammation mice at 1 h post radiotracer injection. White arrows indicated tumors and yellow arrows indicate the inflammatory muscles. **(B, C)** Tumor-to-muscle (T/M) and tumor-to-liver (T/L) ratios among ^68^Ga-NGR-RGD and ^18^F-FDG imaging in SKOV3 **(B)** and ES-2 **(C)** xenograft mice. **(D)** Quantification of ^68^Ga-NGR-RGD and ^18^F-FDG uptake in SKOV3 and ES-2 tumors and inflammatory muscle. **(E)** Tumor-to-inflammatory muscle (Tumor/Inflammatory M) ratios. *p < 0.05, **p < 0.01, ***p < 0.001. Data are expressed as mean ± SD (n = 4).

Next, we performed the PET/CT scan in SKOV3 and ES-2 tumor-bearing mice and turpentine oil-induced muscular inflammation mice using ^18^F-FDG and ^68^Ga-NGR-RGD. As shown in **Figure 2A**, ^68^Ga-NGR-RGD clearly delineated both SKOV3 and ES-2 ovarian tumors, and the tumor contrast of ^68^Ga-NGR-RGD PET imaging was greater than that of ^18^F-FDG. On the contrary, the uptake of ^68^Ga-NGR-RGD in inflammatory muscle was minimal, while avid uptake of ^18^F-FDG in them was observed.

The quantitative data were obtained from the region-of-interest (ROI) analysis. Consistent with the PET images, ^68^Ga-NGR-RGD showed significantly higher tumor-to-muscle (T/M) and tumor-to-liver (T/L) ratios, with values of 2.71 ± 0.21 and 1.05 ± 0.04 for SKOV3-bearing mice and 2.78 ± 0.34 and 1.43 ± 0.16 for ES-2-bearing mice (n=4; all p<0.05), as compared to low T/M and T/L ratios of ^18^F-FDG (0.92 ± 0.22 and 0.92 ± 0.04 for SKOV3, 1.03 ± 0.47 and 0.97 ± 0.26 for ES-2) (**Figures 2B, C**). We also quantified the tracer uptakes in inflammatory muscles and compared them with tumors. The uptakes of ^68^Ga-NGR-RGD in inflammatory muscles were much lower than tumors; however, their ^18^F-FDG uptakes were much higher (**Figure 2D**). And as expected, the tumor-to-inflammatory muscle ratios of ^68^Ga-NGR-RGD in SKOV3 and ES-2 were significantly higher than that of ^18^F-FDG (all p<0.001) (**Figure 2E**).

**Figure 3:**
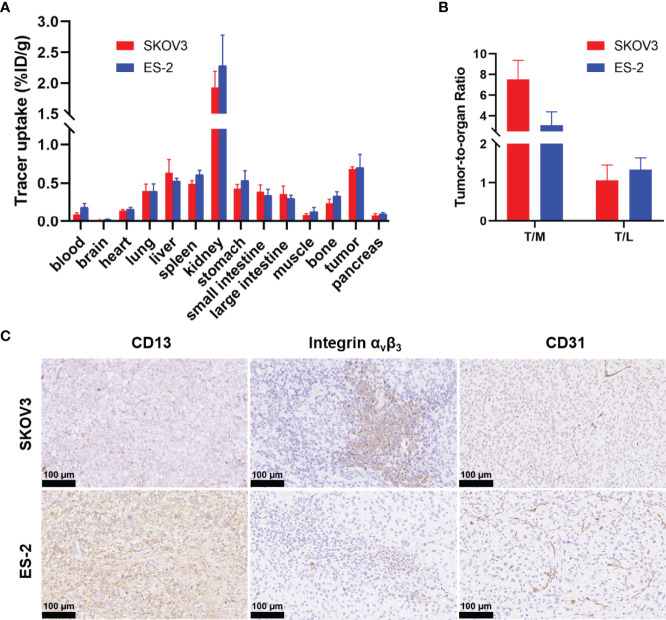
Biodistribution data of ^68^Ga-NGR-RGD in ovarian xenograft mice and immunohistochemistry analysis of tumor tissue sections. **(A)** Biodistribution of ^68^Ga-NGR-RGD in SKOV3 and ES-2 subcutaneous ovarian tumor models at 1 h after injection (n = 4). **(B)** Tumor-to-muscle (T/M) and tumor-to-liver (T/L) ratios of ^68^Ga-NGR-RGD in SKOV3 and ES-2 xenograft mice. **(C)** Immunohistochemistry staining of CD13, integrin α_v_β_3_ and CD31 in SKOV3 and ES-2 tumor sections. Scale bar = 50 μm.

Biodistribution studies of ^68^Ga-NGR-RGD were conducted at 1 h post injection to validate the PET analysis. ^68^Ga-NGR-RGD uptake in SKOV3 and ES-2 xenografts were 0.68 ± 0.03%ID/g and 0.70 ± 0.17%ID/g, respectively (**Figure 3A**). And high tumor-to-muscle and tumor-to-liver ratios were recorded in both ovarian tumors (**Figure 3B**), consistent with PET imaging studies, further indicating the utility of ^68^Ga-NGR-RGD in the diagnosis of ovarian cancer.

### Immunohistochemistry Staining in Tumor Tissues

SKOV3 tumor sections showed high expression of integrin α_v_β_3_ and moderate CD13, and ES-2 tumor sections showed abundant CD13 and moderate integrin α_v_β_3_ (**Figure 3C**). The staining of endothelial marker CD31 (cluster of differentiation 31) was also conducted to evaluate the angiogenesis of tumors. Both SKOV3 and ES-2 tumors displayed neovascularity. Immunohistochemical results of tumor tissues were consistent with western blot results.

### PET Imaging and Biodistribution in Metastatic Models

**Figure 4:**
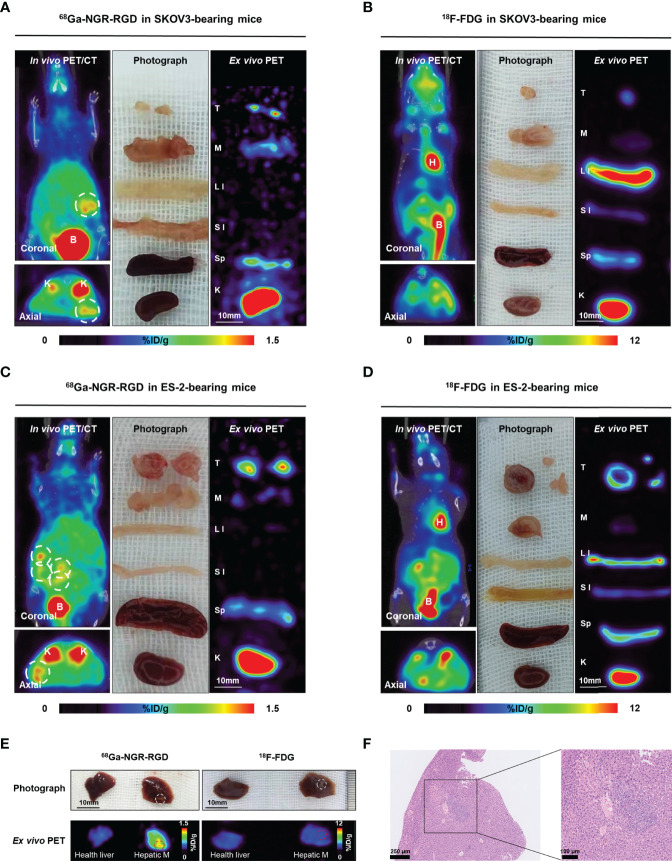
Radiological-surgical correlation of abdominal metastatic models. **(A–D)** Representative static PET/CT images of ^68^Ga-NGR-RGD and ^18^F-FDG in SKOV3 and ES-2 abdominal ovarian metastasis models at 1 h post injection. In ^68^Ga-NGR-RGD PET/CT imaging, several metastatic lesions with strong uptake were found in the peritoneal space [**(A, C)**, white circle]. In ^18^F-FDG PET/CT imaging, there were several stripe high uptake foci **(B, D)**. Surgical exploration was done in the same animal after PET/CT imaging. Diffuse reddish-white nodules with a slightly firm texture were seen in the peritoneal space. Ex vivo PET imaging of excised tissues was performed. The small metastases showed relatively high ^68^Ga-NGR-RGD uptake and low ^18^F-FDG uptake. H, heart; B, bladder; T, tumor; M, muscle; LI, large intestine; SI, small intestine; Sp, spleen; K, kidney. Scale bar = 10 mm **(E)** ES-2 hepatic metastases (Hepatic M) showed strong uptake of ^68^Ga-NGR-RGD, but a similar low uptake of ^18^F-FDG as healthy liver. **(F)** HE staining confirmed that the lesion on liver was tumor tissue. Scale bar = 250 μm or 100 μm.

To further investigate the potential application of ^68^Ga-NGR-RGD in detecting metastases, SKOV3 and ES-2 abdominal metastatic models were established by injecting tumor cells intraperitoneally to simulate peritoneum implantation metastasis of ovarian cancers. As shown in **Figure 4**, peritoneal metastases could be easily delineated from ^68^Ga-NGR-RGD PET/CT imaging in both ovarian tumor models. However, ^18^F-FDG PET showed limited value in detecting these metastatic lesions, which was limited by the relatively low uptake in tumors and high background signals. In the images of ^68^Ga-NGR-RGD (**Figures 4A, C**), several focal uptakes were found in the abdominal space of both SKOV3 and ES-2 group mice, suspected to be the peritoneal implantations; while in the images of ^18^F-FDG (**Figures 4B, D**), several strips with high signal were observed in abdomen. Surgical explorations were done on the same mice after scanning, finding reddish-white nodules with a slightly firm texture in all these groups (**Supplementary Figure 2**). The nodules were extracted for further evaluation, along with muscle, large intestine, small intestine, spleen, and kidney. The nodular tissues (3-6 mm) were confirmed to be ^68^Ga-NGR-RGD-avid but ^18^F-FDG-nonavid by ex-vivo PET imaging. High radioactivity accumulation was found in kidney, indicating that the tracer was mainly excreted *via* kidney. Other tissues in the abdominal cavity such as intestines and spleens showed low signals, indicating that the high signal focuses in PET images were the tumor metastases; whereas, uptake of ^18^F-FDG was high in large intestine, suggesting the high signal regions in the image were not metastases but intestinal physiological uptake. Additionally, there were some liver metastases found in ES-2 abdominal models (**Figure 4E**). The liver metastases of ovarian tumor showed a strong uptake of ^68^Ga-NGR-RGD but a similar low uptake of ^18^F-FDG as healthy liver, which further demonstrated that ^68^Ga-NGR-RGD has excellent metastasis detection efficiency of small peritoneal implants and liver metastases over ^18^F-FDG. The hematoxylin-eosin (HE) staining confirmed that the lesions on the liver were metastatic tumors (**Figure 4F**).

**Figure 5:**
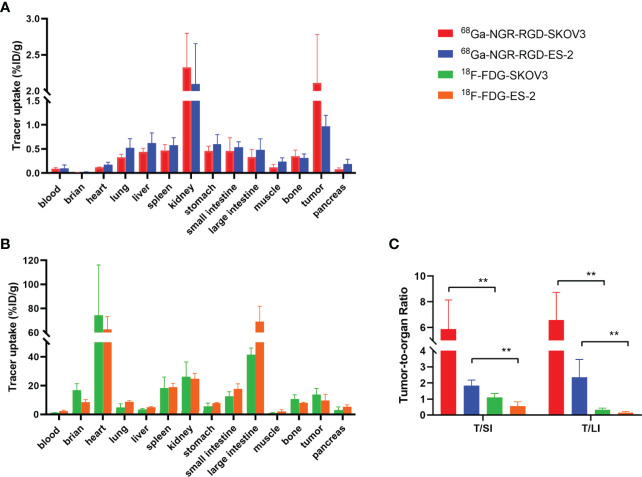
Biodistribution for the validation of PET/CT results. Biodistribution of ^68^Ga-NGR-RGD in SKOV3 and ES-2 **(A)** abdominal metastatic ovarian tumor models and ^18^F-FDG in SKOV3 and ES-2 **(B)** metastatic models at 1 h after tracer injection. **(C)** Metastatic tumor lesions showed avid ^68^Ga-NGR-RGD uptake with a significantly higher tumor-to-small intestine (T/SI) and tumor-to-large intestine (T/LI) comparing to ^18^F-FDG. **p < 0.01. Data are expressed as mean ± SD (n = 4).

Ex-vivo biodistribution studies showed 2.11 ± 0.67%ID/g and 0.97 ± 0.23%ID/g tumor uptake of ^68^Ga-NGR-RGD in the SKOV3 and ES-2 abdominal metastasis models, respectively (**Figure 5** and **Supplementary Table 1**). Tumor uptakes of SKOV3 metastases were higher than SKOV3 subcutaneous tumors, which might be attributed to a better blood supply and the smaller metastasis size (34). Higher tumor-to-muscle (T/M) and tumor-to-liver (T/L) ratios were recorded in ^68^Ga-NGR-RGD group, consistent with the results of s.c. tumor models (**Supplementary Figure 3**). More specifically, tumor-to-small intestine (T/SI) and tumor-to-large intestine (T/LI) ratios of ^68^Ga-NGR-RGD in abdominal metastasis models were significantly higher than that of ^18^F-FDG (p<0.01), which was consistent with PET imaging (**Figure 5C**).

## Discussion

CD13 and integrin α_v_β_3_ are two important angiogenic factors involved in the regulation of tumor angiogenesis and tumor progression and several related targeted tracers were developed for the detection of ovarian cancers, with proven specific and sensitive targeting ability to ovarian cancers (22–25). However, due to the high heterogeneity, and genetical instability of ovarian cancer leading to a progressive increase in the number of different angiogenic factors as the cancer progress to advanced stages (20, 29, 30, 35, 36), the single-receptor targeted imaging strategies may only cover a limited subset of the patients. Owing to the dual-receptor binding property, improved *in vivo* kinetics, and increased circulation half-life, heterodimer tracers are expected to be more sensitive than single receptor-targeted tracers, especially when only one receptor type is overexpressed in a tumor model (31, 37, 38). In this study, we investigated the ability and potential of our recently developed dual CD13 and integrin α_v_β_3_ targeted tracer ^68^Ga-NGR-RGD, as a tumor-specific PET imaging agent, for the early diagnosis and staging of ovarian tumors. ^68^Ga-NGR-RGD exhibited sharp contrasts in subcutaneous ovarian xenografts and metastases, higher tumor-to-background ratios, and in addition, high capability for distinguishing tumor from inflammatory tissue which is superior to ^18^F-FDG, suggesting it has great potential to provide an enhancement to the standard diagnostic imaging of ovarian cancer.

Here, three ovarian tumor cell lines were selected and confirmed to express high level of integrin α_v_β_3_ and/or CD13, indicating the possibility of dual-receptor targeted tracer for the detection of ovarian tumors. *In vitro* studies showed high uptake of ^68^Ga-NGR-RGD in three ovarian cancer cell lines, and blocking studies showed significant decrease tracer uptake, validating the specific binding of our radio-tracer towards integrin α_v_β_3_ and CD13 on ovarian tumor cells. Both SKOV3 and ES-2 subcutaneous metastatic tumors were clearly visualized by ^68^Ga-NGR-RGD PET imaging at 1 h post tracer injection, although SKOV3 cells expressed a high level of integrin α_v_β_3_ but relatively low level of CD13, and ES-2 expressed a high level of CD13 but low integrin α_v_β_3_. These PET imaging results suggested a broad application of ^68^Ga-NGR-RGD in the detection of ovarian tumors with improved tumor-targeting efficacy and sensitivity. Specifically, we could readily identify the location of small peritoneal implants and liver metastases (3-6 mm) in SKOV3 and ES-2 abdominal metastatic models. These results demonstrated the utility of ^68^Ga-NGR-RGD for the sensitive detection of integrin α_v_β_3_ and/or CD13 positive ovarian tumors.

When compared with ^18^F-FDG, greater contrast of subcutaneous and metastatic tumors was observed in ^68^Ga-NGR-RGD PET imaging of SKOV3 and ES-2 ovarian tumor models with significantly higher tumor-to-background ratios (T/M and T/L). In addition, the overall abdominal background uptake of ^68^Ga-NGR-RGD with exception of urinary system was relatively low, so small metastases could be clearly delineated and easily differentiated from background uptake of surrounding tissues. However, ^18^F-FDG accumulated heavily in the large intestine due to physiological intestinal uptake, which often makes it difficult to distinguish between normal intestinal uptake with adjacent abdominal or pelvic tumor or nodal uptake (39–42). Moreover, in turpentine oil-induced muscular inflammatory lesions (**Supplementary Figure 1**), high uptake of ^18^F-FDG was observed in inflammatory cells (neutrophils and macrophages) and granulation tissues, which showed similar histology and FDG-avid features to the reported studies (43–45), mimicking a false-positive lesion of ^18^F-FDG PET. In contrast to ^18^F-FDG, ^68^Ga-NGR-RGD showed low accumulation in inflammatory muscles. Therefore, the false-positive results in physical uptake of surrounding tissues and inflammatory changes detected by ^18^F-FDG can potentially be avoided using ^68^Ga-NGR-RGD as a more tumor-specific imaging agent.

There are many potential applications that probably profit from PET imaging targeting CD13 and integrin α_v_β_3_. With the great contrast of small tumors and higher tumor-to-background ratios than ^18^F-FDG, ^68^Ga-NGR-RGD could provide significant additional information, such as the relationship between tumor lesions with adjacent tissues and distant metastases, for determining TNM staging and optimal treatment options, so it suggests that ^68^Ga-NGR-RGD is a potential candidate to be added to the workup and treatment planning of patients with ovarian tumors. In addition, anti-angiogenesis therapy has been regarded as a new era for tumor treatment in recent years, and targeting the tumor neovascularization, including CD13 and integrin α_v_β_3_, has become a widely accepted therapeutic strategy in clinic (46, 47). The heterodimer strategy may also help with the development of therapy molecules, allowing for selection of responders and treatment response monitoring during and after therapy.

One limitation of this study is that only two types of tumor models were used, while ovarian tumors are highly heterogeneous with complex tumor components (48). Future work will evaluate tumor uptake of ^68^Ga-NGR-RGD in other types of tumor models, especially patient-derived xenograft models, with a various expression of CD13 and integrin α_v_β_3_. Currently, ^68^Ga is usually produced by an in-house ^68^Ge/^68^Ga generator, and one elution could provide a dose enough for 2-5 patients based on the specification of the generator. Therefore, we believe the final cost of a ^68^Ga-tracer scan will be acceptable and should be close to routine ^18^F-FDG PET scans (after considering the cyclotron and its maintenance). Clinical studies evaluating the safety and efficacy of the dual-receptor targeted tracer in humans are ongoing, which will be free of charge for the patients enrolled, and we will report relevant data in the future.

In conclusion, ^68^Ga-NGR-RGD demonstrated a promising application for early diagnosis, staging, and follow-up of ovarian cancer, as it showed high tracer uptake, sharp contrasts in subcutaneous xenograft and metastases, and higher tumor-to-background ratios in ovarian tumor models with different expression levels of CD13 and integrin α_v_β_3_, demonstrating superior diagnostic values than ^18^F-FDG PET/CT. Meanwhile, *in vivo* PET imaging studies showed significantly lower accumulation of ^68^Ga-NGR-RGD in inflammatory lesions as compared to ^18^F-FDG, suggesting the potential of ^68^Ga-NGR-RGD for differentiating between tumor and non-tumor inflammation.

## Data Availability Statement

The original contributions presented in the study are included in the article/**Supplementary Material**. Further inquiries can be directed to the corresponding authors.

## Ethics Statement

The animal study was reviewed and approved by the Institutional Animal Care and Use Committee of Tongji Medical College of Huazhong University of Science and Technology.

## Author Contributions

YL wrote the majority of the manuscript. YL, FS, and HJ performed the canine operations. XS and XYL assisted in PET imaging and biodistribution studies. XX, QL, and YZ provided specific experimental advice and technique support. YG and DZ was the primary developer of NGR-RGD and provided guidance for dosing and imaging decisions. YL, XLL, and YG designed the experiments, analyzed the data, and edited the manuscript. All authors contributed to the article and approved the submitted version.

## Funding

This work was supported by the National Natural Science Foundation of China (81801738, 81630049).

## Conflict of Interest

The authors declare that the research was conducted in the absence of any commercial or financial relationships that could be construed as a potential conflict of interest.

## Publisher’s Note

All claims expressed in this article are solely those of the authors and do not necessarily represent those of their affiliated organizations, or those of the publisher, the editors and the reviewers. Any product that may be evaluated in this article, or claim that may be made by its manufacturer, is not guaranteed or endorsed by the publisher.
