# Supplementary material for: Evaluation of a CD13 and Integrin αvβ3 Dual-Receptor Targeted Tracer 68Ga-NGR-RGD for Ovarian Tumor Imaging: Comparison With 18F-FDG
Source: Front Oncol. 2022 May 18;12:884554. doi: 10.3389/fonc.2022.884554 (PMC9158524; doi:10.3389/fonc.2022.884554)
Supplement: Supplementary file 1 [file DataSheet_1.docx]

Supplementary Material

Supplementary Data
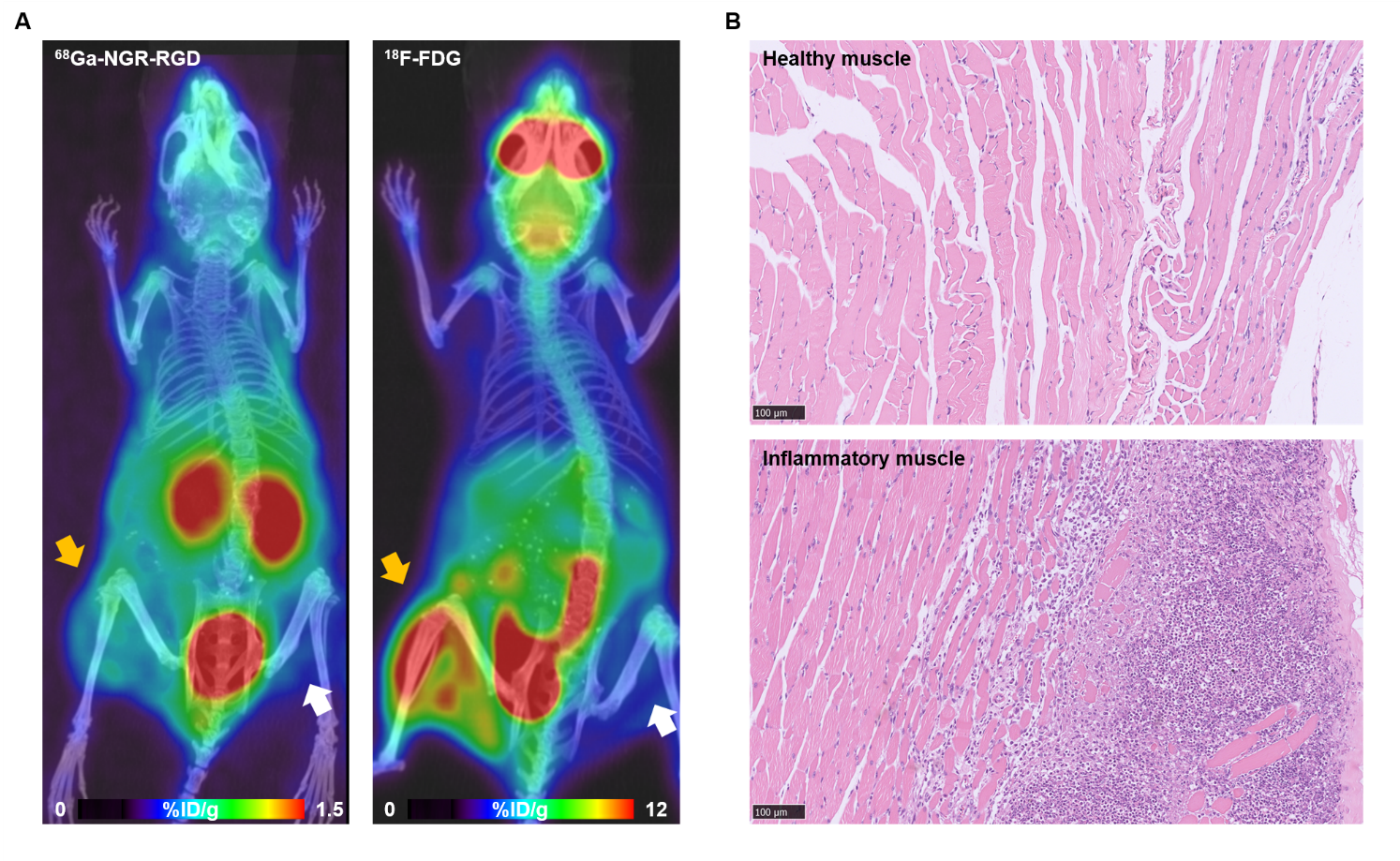


**Supplementary Figure 1.** PET/CT imaging and tissue HE staining changes of muscular inflammation models. (A) PET/CT maximum intensity projection (MIP) images of ^68^Ga-NGR-RGD and ^18^F-FDG in turpentine oil-induced muscular inflammation mice. Yellow arrows indicate the inflammatory muscle; white arrows indicate healthy muscle. (B) HE staining results of healthy and inflammatory muscle. Scale bar = 100 μm.


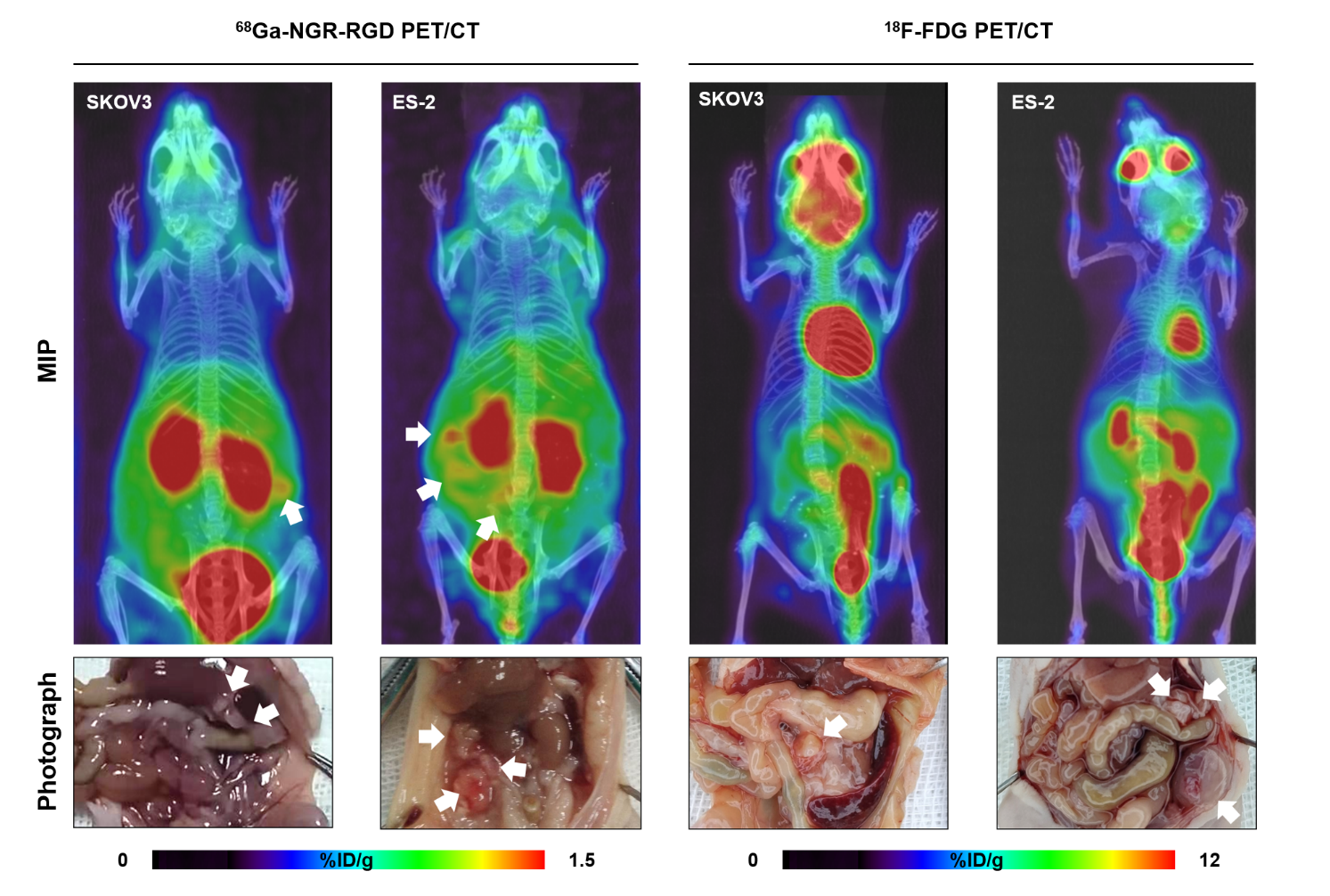


**Supplementary Figure 2.** Radiological-surgical correlation of abdominal metastasis of ovarian cancer. Representative images of ^68^Ga-NGR-RGD and ^18^F-FDG in mice with SKOV3 or ES-2 abdominal metastasis. In ^68^Ga-NGR-RGD PET/CT imaging, several metastatic lesions with strong uptake were found in the peritoneal space. In ^18^F-FDG PET/CT imaging, there are several stripe or sheet high uptake foci. Surgical exploration was conducted after terminal PET/CT imaging. Diffuse lesions were seen at the location of focal uptake on the ^68^Ga-NGR-RGD PET/CT imaging, as well as in the peritoneal space of ^18^F-FDG imaging mice. White arrows indicate the location of metastatic tumors.


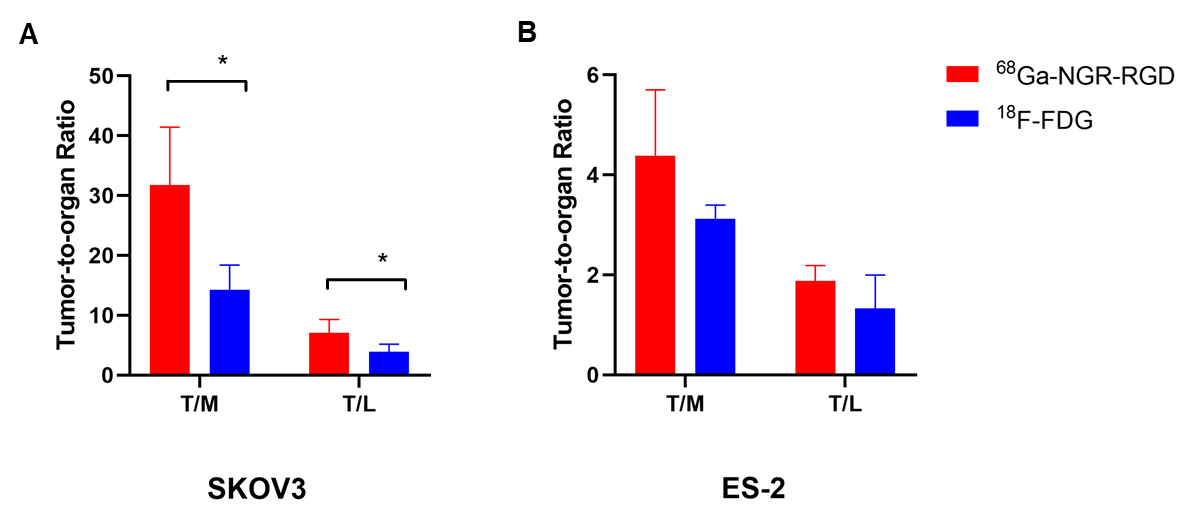


**Supplemental Figure 3**. Tumor-to-muscle (T/M) and tumor-to-liver (T/L) ratios of ^68^Ga-NGR-RGD and ^18^F-FDG in SKOV3 and ES-2 xenograft mice. ^68^Ga-NGR-RGD showed higher tumor-to-background ratios comparing to ^18^F-FDG.

**Supplementary Table 1.** Biodistribution study of abdominal metastatic models immediately after ^68^Ga-NGR-RGD or ^18^F-FDG PET/CT scanning. All data are presented as mean ± SD (n=4).

| Tracer Uptake (% ID/g) | ^68^Ga-NGR-RGD | | ^18^F-FDG | | |
| --- | --- | --- | --- | --- | --- |
|  | **SKOV3** | **ES-2** | **SKOV3** | | **ES-2** |
| Blood | 0.09 ± 0.02 | 0.09 ± 0.08 | 1.06 ± 0.24 | | 2.30 ± 0.79 |
| Brain | 0.01 ± 0.01 | 0.02 ± 0.01 | 16.84 ± 4.52 | | 8.49 ± 1.83 |
| Heart | 0.12 ± 0.01 | 0.17 ± 0.05 | 74.44 ± 41.67 | | 62.61 ± 10.64 |
| Lung | 0.33 ± 0.06 | 0.52 ± 0.19 | 4.97 ± 2.54 | | 8.81 ± 0.86 |
| Liver | 0.44 ± 0.07 | 0.62 ± 0.21 | 3.50 ± 0.72 | | 5.10 ± 0.43 |
| Spleen | 0.47 ± 0.12 | 0.57 ± 0.16 | 18.34 ± 7.62 | | 18.97 ± 2.52 |
| Kidney | 2.33 ± 0.47 | 2.10 ± 0.56 | 26.23 ± 10.16 | | 24.89 ± 3.62 |
| Stomach | 0.46 ± 0.10 | 0.60 ± 0.20 | 5.68 ± 2.26 | | 7.93 ± 0.43 |
| Small intestine | 0.46 ± 0.27 | 0.53 ± 0.11 | 12.20 ± 3.22 | | 17.71 ± 3.62 |
| Large intestine | 0.33 ± 0.16 | 0.48 ± 0.23 | 41.46 ± 4.69 | | 69.15 ± 12.72 |
| Muscle | 0.12 ± 0.07 | 0.24 ± 0.08 | 1.00 ± 0.35 | | 1.98 ± 1.49 |
| Bone | 0.35 ± 0.13 | 0.31 ± 0.08 | 10.67 ± 3.01 | | 8.06 ± 0.53 |
| Tumor | 2.11 ± 0.67 | 0.97 ± 0.23 | 13.73 ± 4.31 | | 9.66 ± 4.32 |
| Pancreas | 0.08 ± 0.03 | 0.18 ± 0.10 | 3.16 ± 2.13 | | 5.33 ± 1.40 |
| Tumor/Small intestine | 5.88 ± 2.26 | 1.83 ± 0.35 | 1.09 ± 0.26 | 0.56 ± 0.27 | |
| Tumor/Large intestine | 6.58 ± 2.15 | 2.35 ± 1.12 | 0.33 ± 0.10 | 0.15 ± 0.07 | |
